# Cellular and clinical impact of protein phosphatase enzyme epigenetic silencing in multiple cancer tissues

**DOI:** 10.1186/s40246-024-00592-x

**Published:** 2024-03-12

**Authors:** Edward Wiltshire, Manuel Castro de Moura, David Piñeyro, Ricky S. Joshi

**Affiliations:** 1 Leicester Cancer Research Centre, Department of Genetics and Genome Biology, Robert Kilpatrick Clinical Sciences Building, Leicester Royal Infirmary, Leicester, UK; 2 Josep Carreras Leukaemia Research Institute (IJC), Badalona, Barcelona, Spain

**Keywords:** Cancer, Epigenetics, DNA methylation, Protein prosphatase enzymes, Transcriptomics, RNA-seq, Hyper-methylation, Gene-silencing, Biomarker

## Abstract

**Background:**

Protein Phosphatase Enzymes (PPE) and protein kinases simultaneously control phosphorylation mechanisms that tightly regulate intracellular signalling pathways and stimulate cellular responses. In human malignancies, PPE and protein kinases are frequently mutated resulting in uncontrolled kinase activity and PPE suppression, leading to cell proliferation, migration and resistance to anti-cancer therapies. Cancer associated DNA hypermethylation at PPE promoters gives rise to transcriptional silencing (epimutations) and is a hallmark of cancer. Despite recent advances in sequencing technologies, data availability and computational capabilities, only a fraction of PPE have been reported as transcriptionally inactive as a consequence of epimutations.

**Methods:**

In this study, we examined promoter-associated DNA methylation profiles in Protein Phosphatase Enzymes and their Interacting Proteins (PPEIP) in a cohort of 705 cancer patients in five tissues (Large intestine, Oesophagus, Lung, Pancreas and Stomach) in three cell models (primary tumours, cancer cell lines and 3D embedded cancer cell cultures). As a subset of PPEIP are known tumour suppressor genes, we analysed the impact of PPEIP promoter hypermethylation marks on gene expression, cellular networks and in a clinical setting.

**Results:**

Here, we report epimutations in PPEIP are a frequent occurrence in the cancer genome and manifest independent of transcriptional activity. We observed that different tumours have varying susceptibility to epimutations and identify specific cellular signalling networks that are primarily affected by epimutations. Additionally, RNA-seq analysis showed the negative impact of epimutations on most (not all) Protein Tyrosine Phosphatase transcription. Finally, we detected novel clinical biomarkers that inform on patient mortality and anti-cancer treatment sensitivity.

**Conclusions:**

We propose that DNA hypermethylation marks at PPEIP frequently contribute to the pathogenesis of malignancies and within the precision medicine space, hold promise as biomarkers to inform on clinical features such as patient survival and therapeutic response.

**Supplementary Information:**

The online version contains supplementary material available at 10.1186/s40246-024-00592-x.

## Background

Protein phosphorylation is a post-translational modification that is vital for controlling signalling pathways and homeostatic maintenance of human cells such as metabolism, transcription/translation and cell division [1]. Protein phosphatases (PPE) are enzymes that catalyse the removal of phosphate groups from cell signalling proteins by hydrolysis, reversing the action of protein kinases (PK). The activities of both are highly coordinated and orchestrate responses from external stimuli and/or relay information for key transcriptional responses that stimulate or inhibit growth and protect the cell which are highly sensitive to small changes in PPE activity [2, 3]. PPE are categorized into two subtypes according to their specificity and action; Protein Tyrosine Phosphatases (PTP) and Protein Serine/Threonine Phosphatases (PSTP). PTP are further divided into (a) receptor-like and non-receptor PTP and (b) Dual-Specificity Phosphatases (DUSP). Typical PTP catalyse phosphotyrosine residues whereas DUSP dephosphorylate phosphoserine/threonine and phosphotyrosine amino acids. PSTP are also divided into two sub-groups; PhosphoProtein Phosphatases (PPP) and Metal-dependent Protein Phosphatases (PPM) [4, 5]. . PPE and PK represent arguably the most studied group of proteins in the literature to date. The uncontrolled suppression of PPE has often been described as a cause of dysregulated cell signalling programs that lead to human disease [3, 6]. In cancer, an imbalance in phosphorylation equilibrium has been reported as a cause of abnormal cell proliferation, dissemination and insensitivity to therapeutic treatment (extensively reviewed in Turdo et al., 2021 [7]). Many PK are well-known oncogenes, therefore PPE that counteract PK are assumed to be tumour suppressors [8]. To date, numerous PPE have been reported with tumour suppressor activity, PTEN as the most documented example. Identified as a tumour suppressor in 1997, *PTEN* was initially observed to be deleted in brain, breast and prostate cancer tissue [9]. Protein Phosphatase 2 A (PP2A) is the most expressed PSTP and described as a tumour suppressor in several cancers, e.g. breast, lung and melanomas [10] due to its role in inhibiting signal transduction pathways such RAF-MEK-ERK and Ras/PI3K/PTEN/Akt/mTOR. These pathways favour a number of cellular functions vital for tumour growth, such as cancer proliferation, reduced sensitivity to apoptotic signals and activation of pro-survival pathways [11, 12]. This dysregulation is essential to create an environment conducive to malignancies. PTP are one of the most recognized group of genes that make up the tumour suppressor family and are frequently inactivated and/or mutated in a variety of cancers [13, 14]. For example, PTPRT and PTPRD negatively regulate the JAK/STAT pathway [15]. PTPRH and PTPRB suppress downstream signalling of PI3K/Akt/mTOR and MEK/MAPK pathways by dephosphorylation of the epidermal growth factor receptor (EGFR) [16] and PTPN13 in non-small cell lung cancer by the phosphorylation control of EGFR and HER2 [17]. As well as the PTP, several DUSP are crucial regulators of MAPK proteins, such as ERK and JNK and therefore considered critical tumour suppressors [18]. Other types of phosphatases not discussed above also have important regulatory roles as tumour suppressors such as cellular prostatic acid phosphatase (PAcP) whose loss of expression leads to prostate carcinogenesis [19] or INPP5K, a phosphoinositide phosphatase gene associated with tumour suppressor activity in endometrial carcinoma [20].

Epigenetic dysregulation has been identified in cancer cells and mainly consists of global DNA hypomethylation with DNA hypermethylation at promoters of specific tumour-suppressor genes resulting in transcriptional silencing [21]. The clinical implications of DNA methylation have been an important characteristic to understand cellular transformation and is an important tool for cancer diagnosis, prognosis and therapy monitoring [22]. In spite of the overwhelming evidence for PPE as tumour suppressors and the role of DNA hypermethylation plays in transcription silencing, only a handful of PPE have been epigenetically characterized in primary tumours [23–30].

Advances in sequencing technologies, data availability and bioinformatic approaches have enabled simultaneous analyses of large sample sizes and -omic datasets. In this study, we performed an exhaustive, systematic analysis of promoter DNA methylation of genes that encode PPE and PPE-interacting proteins (PPEIP) in five different malignant tissues to identify aberrant DNA hypermethylation profiles that are absent in healthy controls (epimutations). We report the frequency of epimutations discerned in Colorectal, Oesophageal, Lung, Pancreatic and Stomach cancers and their effect on transcription, gene regulatory networks/pathways as well as providing examples of their clinical implications such as survival and response to treatment.

## Materials and methods

### Genome-wide DNA methylation array samples

A total of 729 genome-wide DNA methylation array datasets were initially downloaded from publicly available databases and processed in this study. Raw intensity (idats) files produced using the Infinium® HumanMethylation450 (450 K) BeadChip (Illumina) allowed for genome-wide interrogation of 482,000 CpG’s. Raw idat files from 500 primary tumours from The Cancer Genome Atlas (TCGA) legacy database were downloaded. This consisted of 100 samples each from five cancer subtypes; Colorectal cancer (TCGA-COAD and READ), Oesophageal cancer (TCGA-ESCA), Lung cancer (TCAG-LUAD), Pancreatic cancer (TCGA-PAAD), and Stomach cancer (TCGA-STAD). In order to identify cancer-associated changes in DNA methylation, 450 K data from 50 healthy samples (10/tissue type) from each tissue were used as controls in this study. 40 were downloaded from TCGA (10 each from Colorectal, Oesophageal, Lung and Pancreatic tissue) and 10 healthy stomach 450 K datasets were downloaded from Gene Expression Omnibus (GEO) under the accession number GSE127857. Additionally, 450 K data from a second independent test cohort of healthy tissue controls from 47 individuals were downloaded from TCGA (Large intestines (*n* = 16), Oesophagus (*n* = 8), Lung (*n* = 16), Pancreas (*n* = 5), and Stomach (*n* = 2)) and analysed to identify hypermethylation events at PPEIP gene promoters in a population of healthy (non-cancer) tissues. The paucity of healthy tissue where 450 K data is available, limited the test cohort to 47 samples. 204 cancer cell line 450 K datasets were acquired from the Catalogue Of Somatic Mutations In Cancer (COSMIC) database from the Wellcome Trust Sanger Institute as detailed in [31]. Raw intensity files were downloaded corresponding to the same five cancer subtypes as described above; Colorectal (*n* = 49), Oesophageal (*n* = 35), Lung (*n* = 61), Pancreas (*n* = 31) and stomach (*n* = 28) cancer cell lines. Finally, idat files for 25 embedded 3D cultures (organoid) representing the same 5 cancer subtypes were downloaded from GEO under the accession number GSE144213 (Colorectal *n* = 11, Oesophageal *n* = 4, Lung *n* = 1, Pancreatic *n* = 7 and Stomach (*n* = 2) .

### DNA methylation quality control, normalization and filtering

Raw signal intensity values were initially QC’d and pre-processed from subsequent idat files in R statistical environment (v3.6.1) (r-project.org) [32] using minfi Bioconductor package (v1.32.0) [33, 34] and processed in batches by tissue, cancer cell model and healthy samples independently. Quality control steps were applied to minimize errors and remove poor probe signals. Putative labelling errors were ascertained by examining methylation status at sex chromosomes of each individual. Duplicate samples were discerned by the SNP analysis feature in minfi. Vigorous quality control steps were performed on all samples and are detailed in [35]. Briefly problematic probes such as failed probes (detection *p* value > 0.01), cross-reacting probes and probes that overlapped single nucleotide variants within +/- 1 bp of CpG sites were removed. Background correction and dye-based normalization was performed using ssNoob algorithm (single-sample normal-exponential out-of-band). Probes hybridising to Chromosomes X and Y were removed from the final analysis. DNA methylation values for each CpG, represented as β-values with 1 representing fully methylated CpG and 0, fully unmethylated CpG were used for analysis. To further assess sample clusters to identify mislabelled samples based on DNA methylation similarity profiles (cancer vs. healthy controls) in the TCGA primary tumour dataset, robust correlations using multi-step bootstrap resampling and Euclidian distance measures for unsupervised hierarchical clustering were performed using the R package pvclust [36]. Ward.D2 minimum variance method for hierarchical clustering formation was produced using the R package hclust function [37]. Final clusters were plotted using the t-Distributed Stochastic Neighbour Embedding (t-SNE) technique using the R package Rtsne [38, 39]. For this, fifty thousand β values from across the genome in all tissue types were randomly selected in 5000 iterations to assemble and visualize inherent sample similarity. A total of 32 duplicate datasets and mislabelled samples (24 primary tumour and 8 healthy controls) were identified and removed from this analysis. Therefore, 705 cancer samples, 42 healthy controls and 47 healthy test controls were used in the final analysis (a breakdown of all sample ID’s, tissues and cell models used in the analysis is provided in Table 1, Supplementary data S2 and S3). All QC, normalization and filtering steps mentioned above were performed separately for each cancer subtype and cell model. The control samples were ran separately to the tumour samples. Downstream analyses were performed under R statistical environment (v3.6.1).

**Table 1:** Breakdown of the Infinium Human Methylation 450 BeadChip (450 K) samples used and excluded in this study. Initial dataset refers to the number of cancer patients and control datasets downloaded and pre-processed for 450 K DNA methylation analysis. Datasets for analysis is the final number of 450 K patient and control samples analysed in this study. Datasets removed were the number of samples excluded after pre-processing and quality control steps, representing incorrect or mislabeled samples.

Tissue dataset	TCGA cancer	CCL	Organoids	Controls	TCGA healthy test
Dataset source:					
Colorectum	100	49	11	10	16
Oesophagus	100	35	4	10	8
Lung	100	61	1	10	16
Pancreas	100	31	7	10	5
Stomach	100	28	2	10*	2
**Total**:	**500**	**204**	**25**	**50**	**47**
**Datasets used for analysis**:					
Colorectum	95	49	11	10	16
Oesophagus	92	35	4	8	8
Lung	94	61	1	10	16
Pancreas	97	31	7	4	5
Stomach	98	28	2	10	2
**Total**:	**476**	**204**	**25**	**42**	**47**
**Datasets excluded**:	24	0	0	8	0
Colorectum	5	0	0	0	0
Oesophagus	8	0	0	2	0
Lung	6	0	0	0	0
Pancreas	3	0	0	6	0
Stomach	2	0	0	10	0
**Total**:	**24**	**0**	**0**	**8**	**0**

TCGA cancer = Primary tumour samples from The Cancer Genome Atlas data repository, CCL = Cancer Cell Line, Organoids = embedded 3D cultures. Controls = healthy tissue samples used as controls to identify cancer-associated hypermethylation at PPEIP gene promoters. TCGA healthy test = separate subset of healthy tissue samples used to identify non-cancer associated hypermethylation events in PPEIP gene promoters in the healthy population. * = Stomach samples were downloaded from GEO under the accession number: GSE127857 (due to the lack of available samples in TCGA data repository)

### Gene expression profiles

In order to assess the putative consequence of DNA methylation on the expression status of all TCGA individuals that harboured outlier DNA methylation at promoters of Protein Phosphatase Enzymes and interacting proteins (PPEIP) and controls, RNA-seq Fragments Per Kilobase of transcript per Million mapped reads (FPKM) values were downloaded using the R Bioconductor package TCGAbiolinks [40, 41] for TCGA primary tumours and healthy tissues. Transcription profiles were only downloaded from matched patients where both DNA methylation and RNA-seq data was available. For cancer cell lines, gene expression profiles (Transcripts Per Kilobase Million or TPM) were downloaded directly from the Cancer Cell Line Encyclopedia (https://portals.broadinstitute.org/ccle) website. No direct analysis was performed between the two different types of RNA-seq expression counts (FPKM vs. TPM).

### Clinical data

Extensive clinical information for all samples from the TCGA project (primary tumours) were retrieved from TCGA using TCGAbiolinks and integrated into R statistical environment (v3.6.1) for overall survival analyses.

### Data analysis pipeline

A curated list of 726 unique Protein Phosphatase Enzymes and interacting proteins (PPEIP) genes was downloaded from Ensembl (http://www.ensembl.org). PPEIP were defined as genes that encode a protein with previously described phosphatase activity and/or encode proteins that form PPEIP subunits, interact with PPEs, or potentially alter their function. Of the 726 PPEIP genes curated, 203 (28%) lacked a 450 K probe for CpG methylation profiling at its associated promoter region and therefore excluded from this study. The full list of 523 PPEIP genes can be found in Supplementary data S1. Promoter associated probes were defined as 450 K probes that measure CpG DNA methylation levels +/- 1500 bp and 200 bp from the transcription start site, present in the 5’UTR and 1st exon of all PPEIP genes. Probe annotation (such as those located in promoters, CpG island/shores, enhancers, etc.) was provided by Illumina’s 450 K manifest file. Average promoter methylation for each PPEIP gene was calculated in all samples. Cancer-associated epimutations in PPEIP gene promoters were defined as absolute increases of 0.66 average beta value in cancer cell lines and absolute increases of 0.33 average beta value in primary tumour and organoid samples compared to average PPEIP promoter methylation in baseline healthy controls. These cut off values were consistent with previously published studies where hypermethylation in cancer cell lines applied a stringent > 0.66 beta value cut off for cellular homogeneity [31, 42], while for primary tumours and organoids that constitute diverse cell types, the cut off is reduced to 0.33 [43, 44]. Moreover, the epigenetic landscape of organoids is inherently closer to primary tumours than cancer cell lines [35]. A full list of average promoter methylation values for the 523 PPEIP genes in all 705 cancer cases and 42 baseline healthy controls can be found in Supplementary data S9. PPEIP epimutations present in the healthy population were defined as > 0.33 average beta value in test control individuals compared to the average baseline controls. This analysis and all downstream applications were performed using R statistical environment.

### Epimutation tissue distribution and statistical analysis

In an even epimutation distribution (*n* = 5007), one would expect the number of epimutations to be proportional to the number of individuals per tissue set. To test this, we calculated the observed epimutation distribution ratio (OEDR) for each cancer tissue. The equation is shown below. The sample share is the fraction of individuals in each tissue tested for epimutations. Epimutation share per tissue is the fraction of epimutations observed in each tissue and expected to be the same value as the tissue sample share in an even distribution model. The observed epimutation distribution ratio (OEDR) is the tissue sample share / epimutation share. For visualization purposes, the OEDR was converted to log2 values. This was calculated for each tissue.

$${\rm{OEDR}}\, = \,{{\left( {{{n\,{\rm{of}}\,{\rm{tissue}}\,{\rm{samples}}} \over {{\rm{Total}}\,n\,{\rm{of}}\,{\rm{tissue}}\,{\rm{samples}}}}} \right)} \over {\left( {{{{\rm{Observed}}\,n\,{\rm{of}}\,{\rm{Epimutations/tissue}}} \over {{\rm{Total}}\,n\,{\rm{of}}\,{\rm{Epinutations}}}}} \right)}}\} \text{Log2}$$

For Parametric tests, Pearson correlation and unpaired students t-tests were applied and for non-parametric data distributions, Spearmans’ rank correlation and Wilcoxon rank test were calculated. For enrichment analyses, Fishers exact and Chi-squared tests were used. Data normality was assessed using a Shapiro-Wilk test. Linear regression models were used to estimate statistical relationships between DNA methylation and drug sensitivity. Kaplan-Meier plots and Log-rank (Mantel-Cox) tests were used to estimate overall survival (OS) in pancreatic cancer individuals with outlier *PTPRM* expression. Individuals in the top 25% quartiles of *PTPRM* expression were considered as “high expression” and “low expression” the bottom 25% quartile. The extreme 25% quartiles were used as standard cut off points to allow for robust statistical analysis performed through Cox proportional hazards regression models. All statistical analyses were carried out with the R statistical environment (v3.6.1) and p values < 0.05 were considered as statistically significant.

### Pathway and transcription factor binding analysis

Biocarta, KEGG and curated wiki pathway analyses were carried out to highlight cellular features and cancer networks that are putatively affected by hypermethylated PPEIP gene promoters. Transcription factor (TF) gene target examination using Encode data was also performed to inform on TF regulatory networks. All analyses were performed using the R package Enrichr [45].

### Drug sensitivity

IC_50_ Z scores corresponding to drug sensitivity were downloaded from the Genomics of Drug Sensitivity in Cancer database (https://www.cancerrxgene.org/). This repository provides drug response data and genomic markers of sensitivity for 809 cancer cell lines and 198 compounds as part of their GDSC2 data release [31]. All cancer cell lines pertinent to this study were downloaded and processed according to tissue type.

A simplified overview of the analysis workflow is provided in Supplementary Fig. 1.

## Results

### Pan-cancer promoter hypermethylation analysis of protein phosphatase enzymes and interacting proteins

**Fig. 1:**
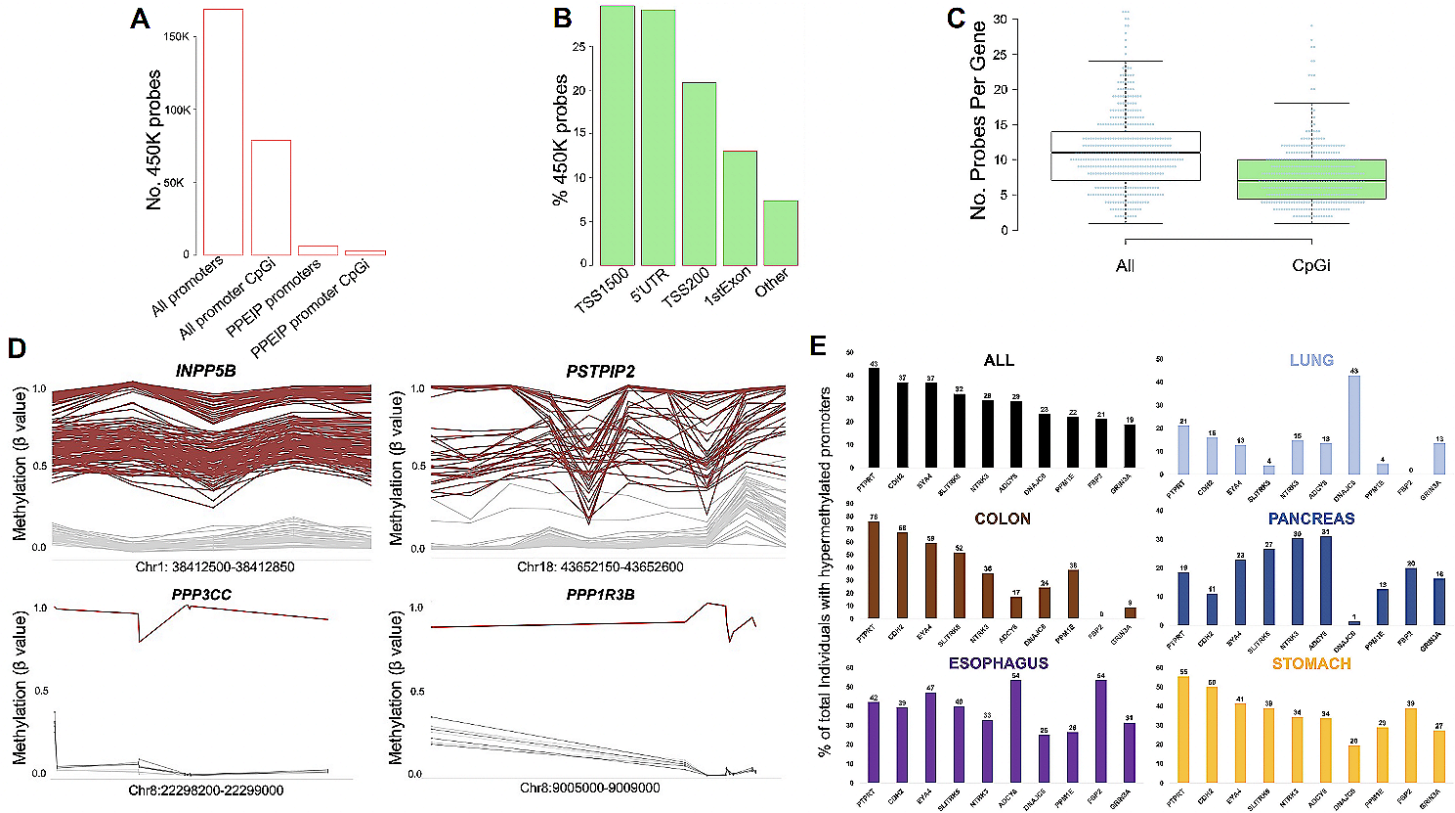
Infinium Human Methylation 450K BeadChip (450K) probe distribution and promoter hyper-methylation analysis reveals cancer-associated epimutations occur with varying frequencies in multiple tissues. **A**. Distribution of 450K probes across all gene promoters (and CpG island-specific) and PPEIP promoters (also showing CpG island specific probes). **B**. % of promoter CpG genomic annotations. TSS1500 = CpG present within 1500 bp from transcription start site (TSS), TSS200 = within 200 bp of TSS, 5’UTR = CpG is found within the 5’ untranslated region of the PPEIP and 1stExon = CpG found in the first exon of the PPEIP gene. The annotation “others” refers to promoter CpG’s that annotate to other genomic regions (such as gene bodies or introns) of overlapping genes. **C**. Distribution of promoter probes per gene annotated to both CpG islands and non-CpG islands. **D**. Examples of hyper-methylated PPEIP promoters for both recurrent (observed in > 5% of the cancer population) and rare (< 1% % of the cancer population) epimutations. Red lines present individuals with outlier epimutations and grey lines, healthy controls. **E**. Bar graph showing the ten most epimutated PPEIP genes. Numbers on the y-axis (and above each bar) represent the % of total cancer individuals in all cancer tissues (ALL) as well as a breakdown of epimutations identified in individuals of specific cancer related tissue

The Infinium HumanMethylation450 BeadChip (450K) platform allows for genome-wide CpG methylation analysis at 485,000 CpG’s located at various genomic regions including 98% of all promoters in refseq-annotated genes. In order to dispel potential probe representation issues, we first analysed the distribution of probes at PPEIP promoters against all other 450K represented genes. 6296 450K probes informed CpG DNA methylation levels in PPEIP promoters (12 probes per gene) and 3260 (52%) of those, in CpG islands associated with PPEIP promoters (6 probes per CpG island) (Fig. 1A). In comparison, 168,664 probes were annotated to all gene promoters (9 probes per gene) and 79,008 (47%) to all promoter associated CpG islands (4 probes per gene) indicating a higher representation of probes for PPEIP gene promoters compared to all other gene promoters (Fig. 1A). The definition of “promoter probes” were those probes that provided semi-quantitative methylation values for CpG’s located within 1500 bp and 200 bp of the transcription start site (TSS1500, TSS200), 5’ untranslated region (5’UTR) or 1st exon of each gene. The majority of PPEIP gene promoter probes were found in the TSS1500 (30%) and the least in the 1st exon (13%) (Fig. 1B). Half of all PPEIP probes were distributed between the 5’UTR (29%) and the TSS200 (21%) (Fig. 1B). The median number of probes per gene was 11, and 7 when considering only CpG islands in PPEIP promoters (Fig. 1C). The Protein Tyrosine Phosphatase gene *PTPMT1* was the PPEIP with the most probes associated with its promoter (*n* = 60).

### PPEIP promoter hypermethylation profiles reveal distinct tissue susceptibility and frequency associated with cancer

Overall, 5007 hypermethylated Protein Phosphatase Enzymes and Interacting Proteins (PPEIP) gene promoters (epimutations) were identified in 593 cancer samples (84%), a median of 4 cancer-associated epimutations per individual (Supplementary Fig. 4B). The distribution of epimutations across samples was significantly imbalanced (*P* = 2.2 10^− 16^). Stomach cancer patients demonstrated the highest percentage of PPEIP epimutation distribution of 27% however, stomach cancer cases only made up 18% of the total sample number (Supplementary Fig. 2A). This represented the highest ratio of epimutation vs. sample share, or observed epimutation distribution ratio (OEDR) of 0.59 (expected OEDR = 0) (Supplementary Fig. 2B). Lung cancer patients showed with the lowest epimutation share with 9%, although 22% of all samples were from lung malignancies (-1.25 OEDR) (Supplementary Fig. 2A and B). Colorectal, oesophageal and stomach cancers showed positive OEDR (0.59–0.25) while lung and pancreatic cancer patients showed negative OEDR (-1.25 and − 0.68 respectively) (Supplementary Fig. 2B). 593 cancer patients (84%) presented with at least one hypermethylated PPEIP promoter in 160 (31%) PPEIP genes analysed as compared with healthy controls. Oesophagus represented the cancer tissue with the highest number of individuals with at least one epimutation (98%) (Supplementary Fig. 3A). This slight inconstancy with the OEDR stomach cancer data can be explained by the individual epimutation frequency in both tissues. 44% of all epimutations detected in the top 10% of individuals with the most epimutations accumulated were attributed to stomach cancer patients compared to only 28% in the oesophagus (Supplementary data S4). This suggests that a subset of stomach cancer patients accumulate higher quantities of epimutations in fewer individuals as compared with oesophageal cancer patients where epimutations are procured less inter-individually but more consistently across individuals. Lung cancer patients presented with the least number of epimutations (67%) consistent with OEDR data (Supplementary Fig. 3A). 96% of all organoid samples harboured at least one hypermethylated PPEIP promoter compared to 86% and 77% of all cancer cell lines and primary tumours respectively (Supplementary Fig. 3B). To assess the data further, we applied our epimutation detection pipeline to 450 K data from a separate test cohort of 47 control individuals presenting the five analysed tissues to identify epimutations in a population of healthy individuals. 11 individuals (23%) were identified carrying 79 hypermethylated PPEIP promoters as compared to 84% in cancer samples. A median epimutation count of 0 per individual was observed (Supplementary Fig. 4A) as compared to 4 in the same tissues in a cancer context (Supplementary Fig. 4B). Epimutations we detected were overwhelmingly enriched in cancer patients as compared to healthy individuals (*P* = 7.02 × 10^− 18^). Interestingly, epimutations detected in healthy samples were only observed in two tissues (Oesophagus *n* = 7 and Pancreas *n* = 4) (Supplementary data S5). This gave us confidence that the epimutations we identified through our bespoke bioinformatic pipeline were cancer-associated.

Next, we examined the frequency of PPEIP-associated epimutations in cancer patients. 5007 epimutations were detected in 160 PPEIP genes (Supplementary data S6). Of the 160 PPEIP genes, 88 (55%) were considered “rare” (observed in < 1% of all cancer cases) and 41 (26%) as “recurrent” (identified in > 5% of cancer samples) (Supplementary data S6). Many recurrent genes were known tumour suppressors with previously described cancer-related promoter hypermethylation anomalies (*PTPN13*, *DUSP5*, [2] *PPP1R14A* [26], *PPP1R3C* [27], *PTPRM* [28] and *IGFBP3* [46–48] validating the robustness of our approach. We also detected several PPEIP genes with previously undescribed recurrent methylation changes (Fig. 1D). INPP5B (Inositol Polyphosphate-5-Phosphatase) is an anti-apoptotic protein with a proliferative role in different cancer types [49, 50] and epimutations were observed in 130 individuals (Fig. 1D) in all 5 tissues (Supplementary Fig. 5). Proline-serine-threonine phosphatase interacting protein 2 or *PSTPIP2* promoter hypermethylation was observed in 37 cases (Fig. 1D) in all 5 tissues examined (Supplementary Fig. 6). PSTPIP2 is required for correct cell cycle function and dysregulation of PSTPIP2 contributes to abnormal proliferation and terminal differentiation in megakaryocytes [51]. In addition to recurrent differentially methylated promoters, several patients displayed rare, previously undescribed epimutations at PPEIP promoters. For example, an epimutation was detected at the promoter of *PPP3CC* (Protein Phosphatase 3 Catalytic Subunit Gamma) in 1 individual (colorectal cancer). PPP3CC repression has been reported to contribute to invasion and growth of glioma cells [52]. Loss-of-function genetic mutations in *PPP1R3B* gene have been associated with lung cancer [53], similarly, DNA methylation associated transcription silencing mimics loss-of-function properties. We observed one individual with oesophageal cancer phenotype harbouring an epimutation at the *PPP1R3B* promoter (Fig. 1D). Beta values for each probe and individuals (cancer and healthy controls) are provided in supplementary data S7.

We examined highly epimutated PPEIP genes and their described roles in human malignancies. Collectively, *PTPRT*, *CDH2*, *EYA4*, *SLITRK5*, *NTRK3*, *ADCY8*, *DNAJC6*, *PPM1E*, *FBP2* and *GRIN3A* were identified as those genes where most cancer individuals were observed to harbour hypermethylated PPEIP promoters. An even distribution of epimutation count was not observed across all cancer tissue types, consistent with the OEDR data. A breakdown of this is presented in Fig. 1E. *PTPRT* was the most epimutated PPEIP with 43% of all cancer cases showing epimutations in this gene. PTPRT is a tyrosine phosphatase with a previously described role as a tumour suppressor in colorectal cancer [13]. Interestingly, the authors also demonstrated that the most frequent genetically mutated tyrosine phosphatase gene was *PTPRT* in their colorectal cancer (CRC) cohort. In line with this finding, *PTPRT* was also the most epimutated gene in our CRC cohort; 76% of all CRC patients carried an epimutation. On the contrary, only 21% and 19% of all lung and pancreatic cancer patients respectively carried an epimutation in *PTPRT* (Fig. 1E). Several of the highest epimutable genes have disparate prevalence of hypermethylated promoters between cancer tissue types. Eyes absent 4 (EYA4) is a threonine-tyrosine phosphatase [54] previously described as a tumour suppressor in multiple cancers examined in this study (CRC [55], oesophagus [56], lung [57] and pancreas [58]). EYA4 promoter DNA methylation has been reported to be negatively correlated with gene expression and plays an important role in cell proliferation inhibition via Wnt and MAPK signalling pathways [59]. The frequency of epimutation is highly contrasting as 59% of CRC patients carry hypermethylated EYA4 promoters compared with only 13% of lung cancer patients (Fig. 1E). Another example is the NTRK3 gene. NTRK3 has been described in important cancer related pathways that promote both survival and cell proliferation and so its role as an oncogene [60] and tumour suppressor [61] is not unexpected. We observed at least a twofold difference in individuals with NTRK3 hyper-methylated promoters between lung cancer (15%) and the other 4 cancers (CRC; 36%, stomach; 34%, Oesophagus; 33% and Pancreas 30%) (Fig. 1E).

### Pan-cancer promoter hypermethylation of PPEIP affect cellular pathways and networks that favour tumour success

**Fig. 2:**
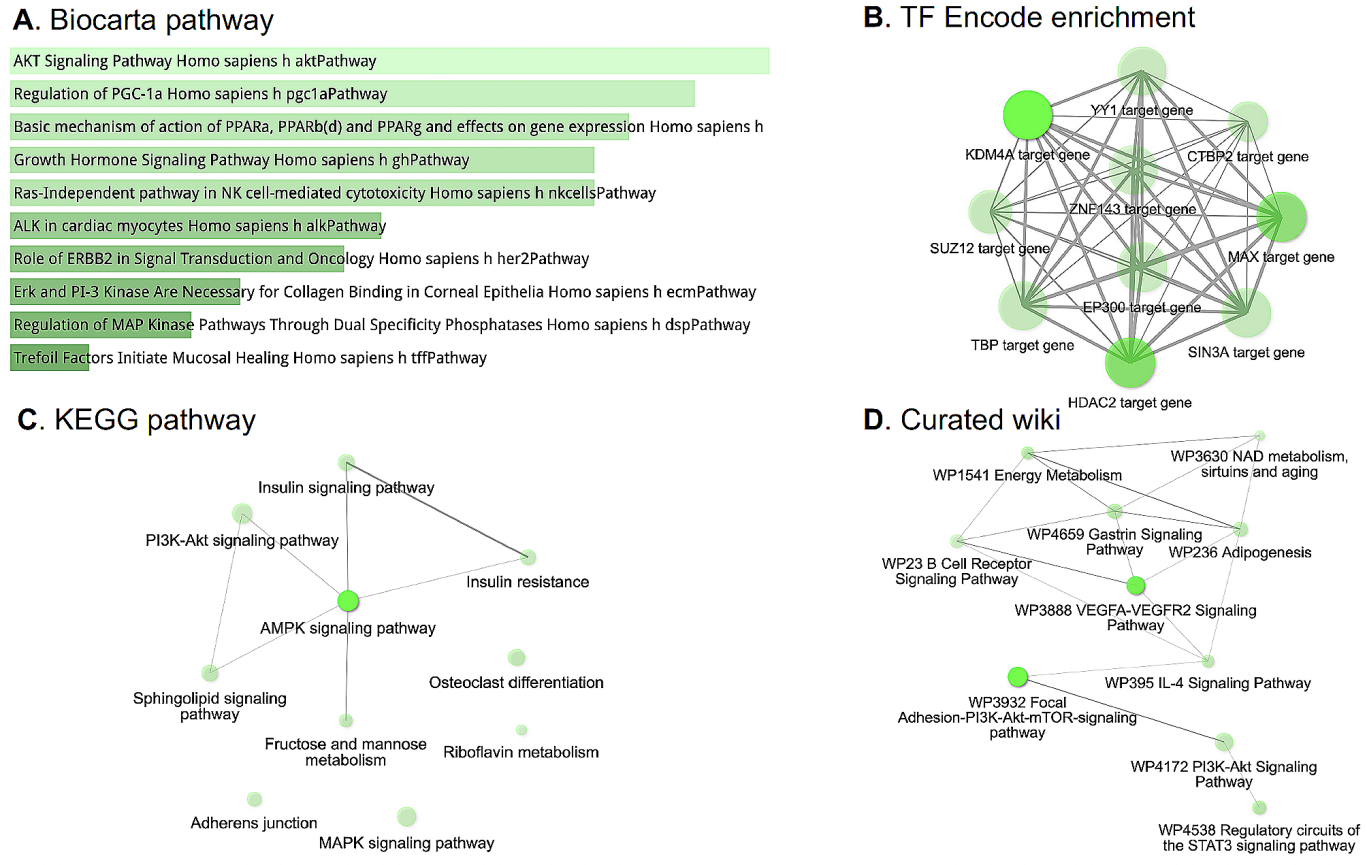
PPEIP genes with hypermethylated promoters are involved in several cancer-associated cellular pathways and mechanisms. (**A**) Biocarta pathway analysis for pathway gene enrichment. The shade of green presents the significance of the specific gene-set or term. (**B**) Enrichment of transcription factor (TF) binding associated with hyper-methylated PPEIP genes from ChIP-seq experiments of > 300 TFs from the ENCODE project. Bright green presents higher number of genes. **C-D**. Orthogonal cellular pathway analyses from two independent resources; Kyoto Encyclopedia of Genes and Genomes (KEGG) and Curated wiki. For Figures A-D, the brighter the tone of green, the more significant the term is. In all network images (**B-D**), the grey lines represent gene content similarity

To further decipher the role of the 160 cancer-associated promoter hypermethylation susceptible PPEIP genes, we performed an enrichment analysis for gene networks, cellular pathways and transcription factor (TF) binding (Fig. 2). For gene network and cellular pathway analysis, three highly cited software were used; Biocarta [62], Kyoto Encyclopedia of Gene and Genomes (KEGG) [63] and curated WikiPathways [64]. All three software demonstrated high overlap of well-known pathways described in cancer cells such PI3K-AKT [65], MAPK [66] and cellular metabolism [67] (Fig. 2A,C,D). All three present actionable targets for anti-cancer drugs [68–70]. Other interesting pathways include angiogenesis related VEGFA-VEGFR2 signalling [71], regulatory circuits of STAT3 signalling pathways [72] as well as gene networks involved in aging [73]. We also interrogated transcription factor (TF) targets computed from ChIP-seq data from the ENCODE project [74]. The genes most affected by promoter hypermethylation are also targets for TF that are highly mutated in cancer such as chromatin remodelers (EP300, HDAC2, KDM4A among others) (extensively reviewed in [75]), cell cycle regulator (SIN3A [76]) and cell proliferation (YY1 [77]) (Fig. 2B).

### Pan-cancer examination of epimutations in protein tyrosine and dual specific phosphatases exhibit aberrations in transcriptomic profiles affecting key cellular networks related to cancer

**Fig. 3:**
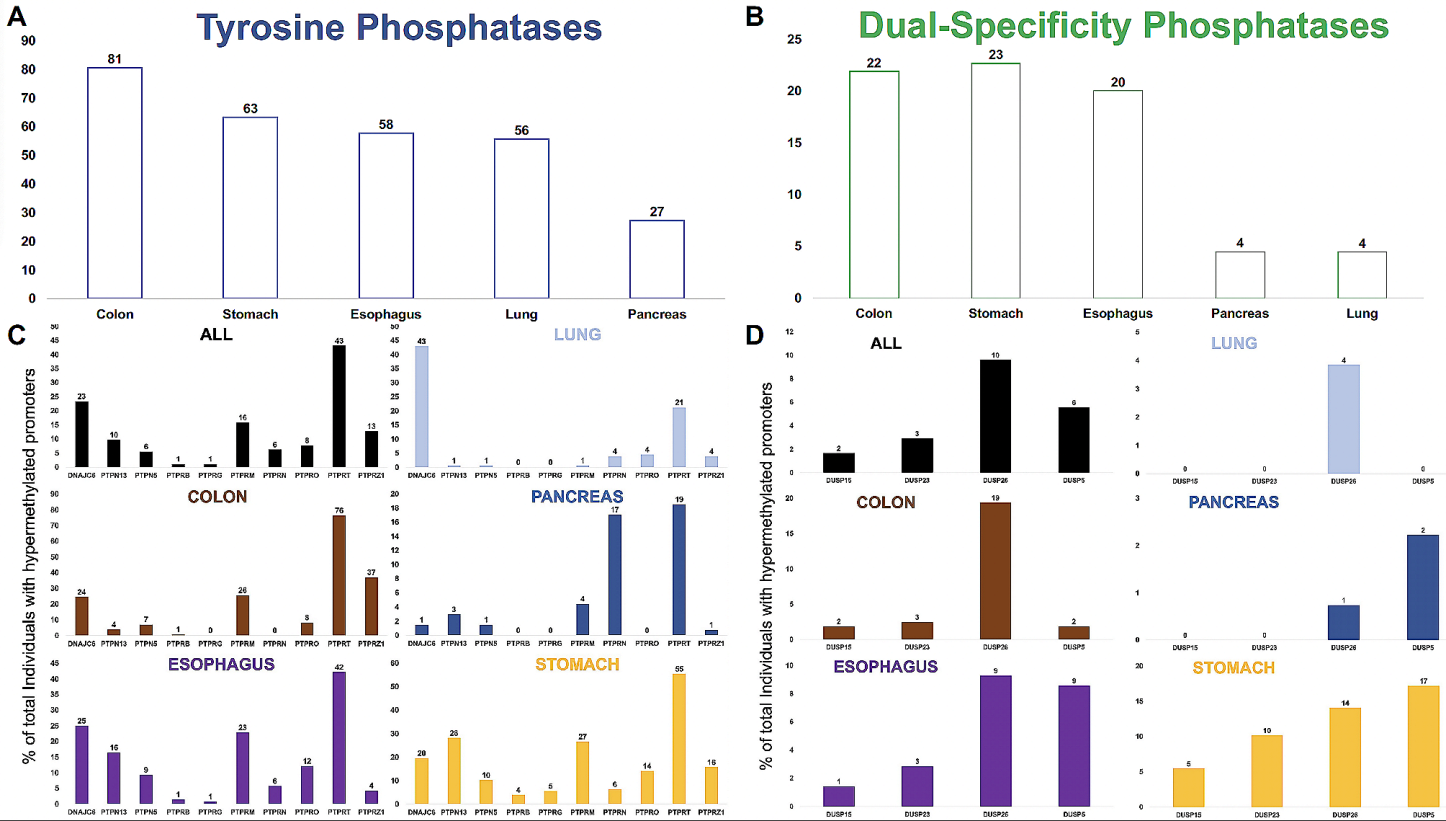
Cancer-associated epimutations are commonly observed in Protein Tyrosine (PTP) and Dual-Specificity Phosphatase (DUSP) gene promoters. **A-B**. Bar graph demonstrating the % of individuals in each cancer subtype that carry at least one cancer-specific epimutation in tyrosine (**A**, blue) and dual specificity (**B**, green) phosphatase gene promoters. **C-D**. Bar graph representing the most ubiquitous PTP (**C**) and DUSP (**D**) gene promoters that harbour cancer-specific hyper-methylated promoters. Numbers above each bar represents % of cancer individuals with epimutations in the genes highlighted on the x-axis. The genes represented in (**C**) and (**D**) were detected in at least 5 individuals

A number of Protein Tyrosine Phosphatases (PTPs) have been described in human cancers as tumour suppressor genes [2] and among the most studied include *PTPRM*; [78, 79], *PTPN13*; [17] and PTPRG; [80]. Therefore, in primary tumours, PTPs would represent a subset of key genes where the effects of promoter DNA methylation-induced transcriptional silencing are detrimental. In this regard, we closely examined PTPs for methylation sensitivity in primary tumours, cancer cell lines and 3D embedded cell cultures (organoids). We also performed the same analysis in dual-specificity phosphatases (DUSP) given their activity and role in cancer [81]. Only few individuals presented hypermethylated promoters for serine / threonine phosphatases (< 2%) and therefore we focused our attention on PTP and DUSP genes. A list of PTPs and DUSPs was compiled from Ensembl and DNA methylation profiles generated for 43 PTP and 24 DUSP genes (Supplementary data S8). 17 PTP (40%) and 7 (29%) DUSP were observed with hypermethylated promoters in 410 (57%) and 102 (14%) cancer cases respectively. Colorectal cancer (CRC) patients demonstrated the highest number of PTP epimutations (81%) and pancreatic cancer patients the lowest (27%) (Fig. 3A). Stomach cancers presented the highest number of individuals with DUSP epimutations (23%) with lung and pancreatic malignancies the least (4%) (Fig. 3B). Further analysis into the role of PTPs and DUSP revealed PTPRT is the most ubiquitously epimutated PTP, 43% of all cancer individuals showed hypermethylated PTPRT promoters. DNAJC6 (23%) and PTPRM (16%) were the second and third most epimutated PTP. PTPRT was also found to be the most epimutated PTP in 4 of the 5 cancer tissues analysed (CRC = 78%, Stomach = 55%, Oesophagus = 42% and Pancreas = 19%) and second most epimutated in Lung (21%). DNAJC6 was the most epimutated PTP in Lung (43%) (Fig. 3C). CRC, stomach and oesophageal cancer showed overall high levels of individuals with PTP hypermethylated promoters. Of the top 10 most epimutated PTPs, Stomach (9/10), Oesophageal (7/10) and CRC (6/10) showed > 5% of individuals with epimutations in PTPs. Pancreas and Lung (2/10) presented with low epimutated PTPs (Fig. 3C). To a lesser extent, DUSP genes were also found to be highly epimutated (Fig. 3B). DUSP26 was the gene with the highest number of individuals with hypermethylated promoters (10%), with DUSP5 (6%), DUSP23 (3%) and DUSP15 (2%) also showing epimutations (Fig. 3D). CRC individuals showed the highest number of epimutated DUSP26 (19%) followed by Stomach (14%) and oesophagus (9%). Again, lung and Pancreas showed the least (4% and 1% respectively). DUSP5 was the most epimutated DUSP in Stomach (17%), Pancreas (2%) and equal to DUSP26 in Oesophageal cancer (9%) (Fig. 3D).

**Fig. 4:**
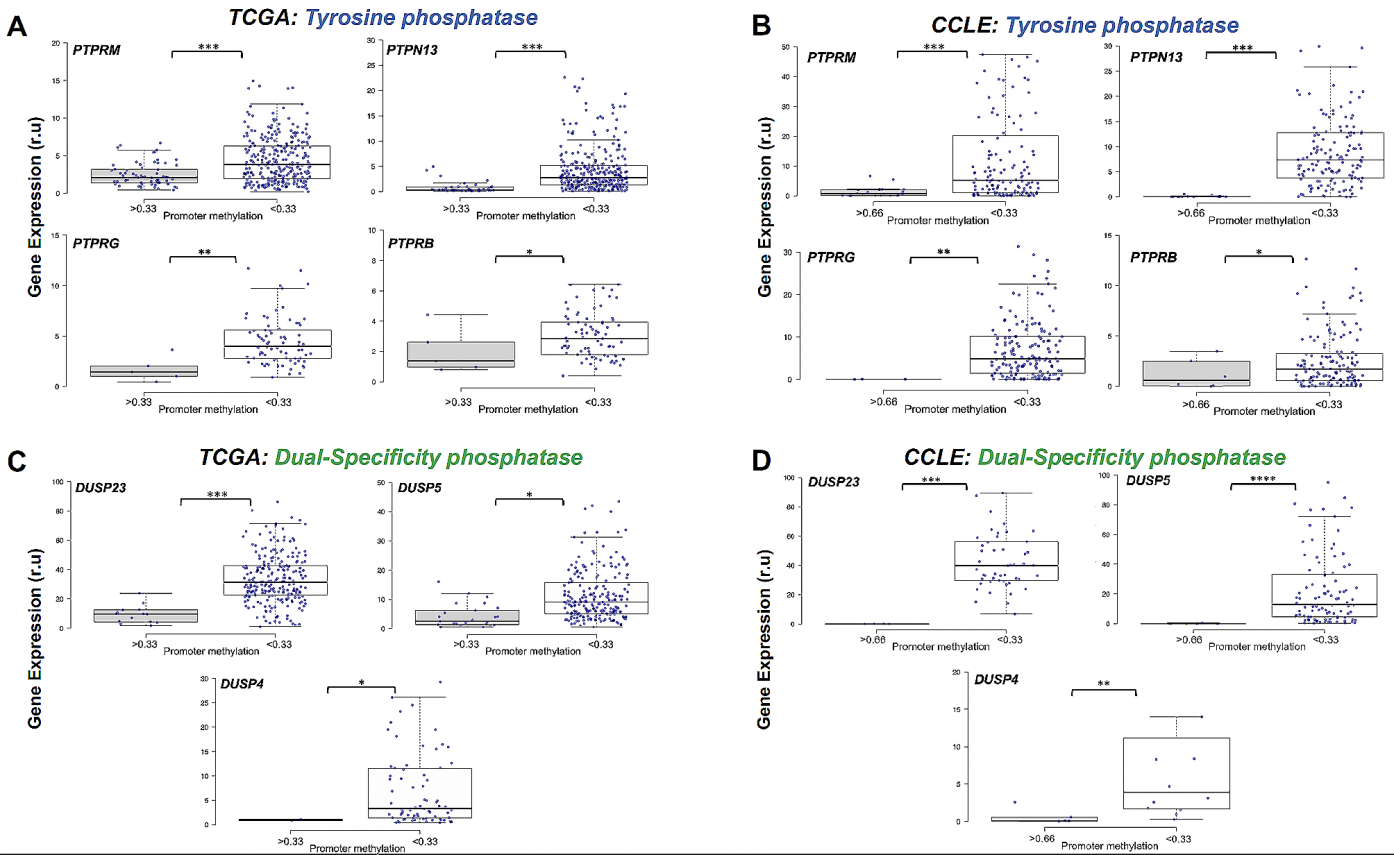
Cancer-specific promoter DNA hypermethylation in Protein Tyrosine (PTP) and Dual-Specificity Phosphatase (DUSP) are associated with gene expression silencing. **A-B**. Boxplots representing the effect of hypermethylated promoters of PTP genes in primary tumours (**A**) and cancer cell lines (**B**). **C-D**. Boxplots representing the effect of epimutations discerned in promoters of DUSP genes from primary tumours (**C**) and cancer cell lines (**C**). Primary tumour and cancer cell line gene expression data was downloaded from The Cancer Genome Atlas (TCGA) and the Cancer Cell Line Encyclopedia (CCLE) projects. Gene expression values are presented as relative units (r.u) and are specific to each project (TCGA = FPKM and CCLE = TPM). Gene names shown at the top left-hand corner of each boxplot and blue dots presents individual (and cancer cell lines) expression values where DNA methylation profiles were also available. *P < 0.05; **P < 0.01; ***P < 0.001

Next, we analysed the effect of epimutations on PTP and DUSP gene expression given not all are expressed in the 5 tissues. 17 PTP and 7 DUSP were detected to contain at least one cancer-associated hypermethylated promoter. An initial analysis of gene expression in normal tissues was conducted using the GTEx portal (gtex.org, [82]) in the five tissues analysed in this study to determine the potential effect of cancer-associated epimutations on PTP and DUSP transcription. 9 of 17 PTPs and 5 of 7 DUSP were observed to be expressed at high levels in at least one healthy tissue type (> 5 TPM) (Supplementary Fig. 7A and B). Further investigation revealed that 4 PTP (PTPRM, PTPN13, PTPRG and PTPRB) and 3 DUSP (DUSP23, DUSP5 and DUSP2) were ubiquitous epigenetic outliers in all cancer cell models, highly expressed in at least one normal tissue (> 5 TPM) and maintained expression in their malignant counterpart prior to segregation based on promoter DNA methylation (Supplementary Fig. 8). Expression profiles from primary tumours (TCGA) and cancer cell lines (CCLE) for the 4 PTP and 3 DUSP are presented in Fig. 4. In each boxplot, gene expression is partitioned by individuals with hypermethylated promoters (> 0.33 average promoter beta value in TCGA and > 0.66 in CCLE vs. healthy controls) (see methods for details). A significant negative correlation between promoter methylation and gene expression was observed in all genes and cell models. We expect these data to also be representative of cancer organoid cell models [35]. Although hypermethylated promoters negatively correlate with gene expression in our cancer cohort (Fig. 4), one exception was observed in the gene *DNAJC6*, where an increase in promoter methylation was positively correlated with gene expression (Supplementary Fig. 9A and C). *DNAJC6* is the second most epimutated gene in all cancer samples and the most epimutated gene in the lung cancer cohort (43%) (Figs. 1 and 3). Although a high number of epimutations were identified in our analysis cohort, several showed very low expression in their pertinent tissues. As mentioned above, PTPRT is the most epimutated gene found in this study however its expression is extremely low (or non-existent) in the 5 tissues analysed (Supplementary Fig. 9B and D) with the highest expression observed in brain tissue (Supplementary Fig. 7A). This interesting finding demonstrates that epigenetic dysregulation in cancer cells occurs independent of an active transcriptional program and may provide important genomic information for other tissues.

### PTPRM epigenetic transcription silencing correlates with poor clinical outcome and reduced anti-cancer drug sensitivity

The role of PTP as tumour suppressors has been described in great detail in a number of tissues [2] and are targets for anti-cancer therapy [83]. In our cancer cohort, PTPRM represented the PTP with highest number of individuals with epimutations and high median expression values in healthy subjects (> 5 TPM). Having demonstrated the presence of *PTPRM* promoter hypermethylation associated transcription silencing (Fig. 4A and B), we studied if PTPRM epigenetic loss in cancer patients had any impact on the clinical outcome in these patients. For this, we leveraged complete clinical and transcriptomic data from all individuals used in this study available in TCGA data repository. We identified that *PTPRM* gene silencing (top 25% quartile expression vs. bottom 25%) in pancreatic cancer patients showed a significant association with poor overall survival probability (Log Rank P = < 0.05, hazard ratio [HR] = 1.82; 95% confidence interval [CI] = 0.995–4.336; P = < 0.05).

**Fig. 5:**
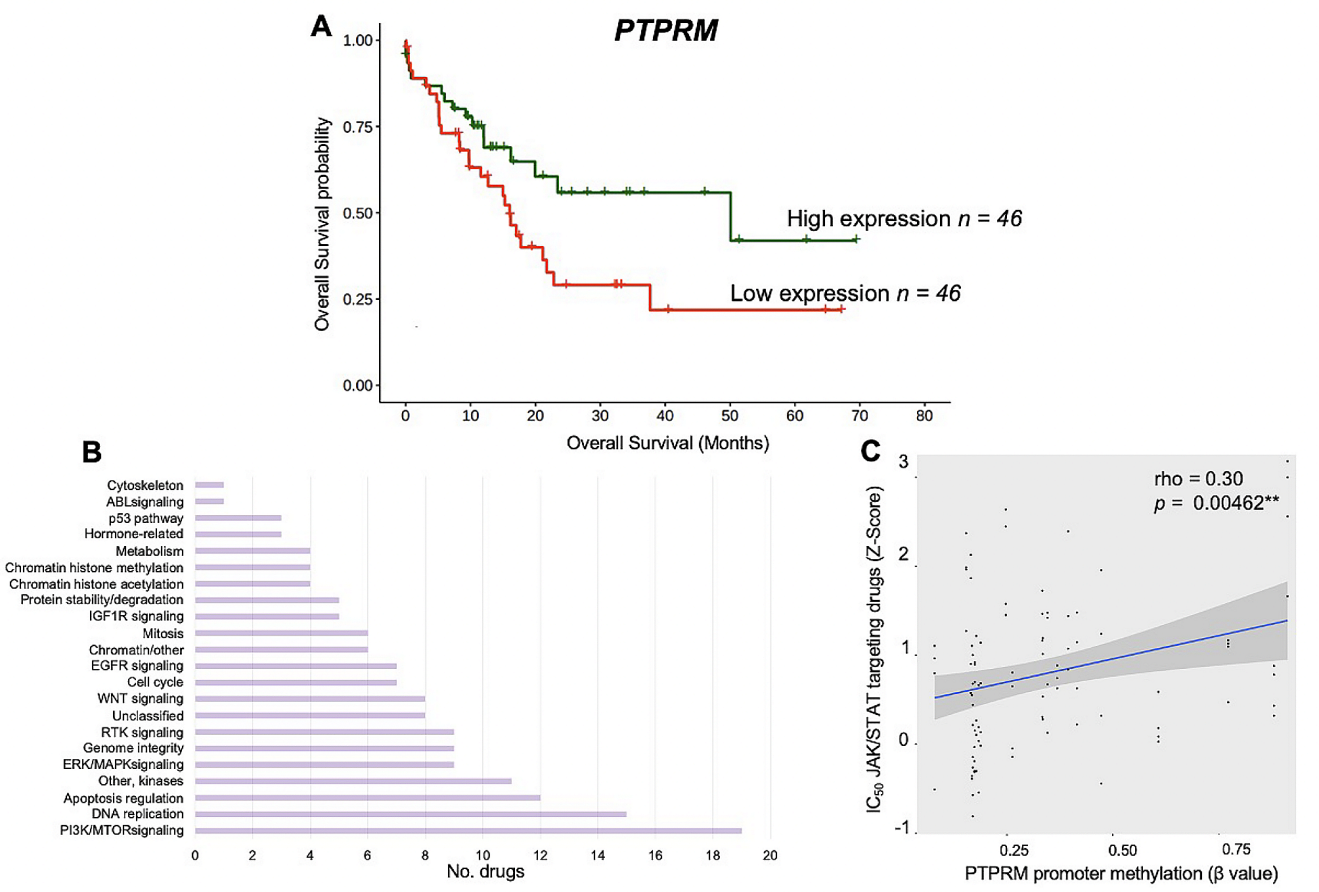
*PTPRM* reduced expression via epigenetic silencing is associated with poor survival and reduced sensitivity to JAK/STAT targeted anti-cancer therapy in pancreatic cancer patients. (**A**) Kaplan–Meier curve showing reduced expression of PTPRM in patients with pancreatic cancer was significantly associated with shorter overall survival, Log Rank P = < 0.05 (HR = 2; 95% CI = 0.757–4.336; P = < 0.05). Green line represents patients in the top 25-percentile of PTPRM expression and the red line, bottom 25-percentile *PTPRM* expression in the pancreatic primary tumour cohort. (**B**) Horizontal bar graph demonstrates the cellular pathways targeted by anti-cancer drugs from GDSC2 (Genomics of Drug Sensitivity in Cancer) project. Drugs targeting “other” category were excluded. (**C**) Scatter plot illustrating IC_50_ concentration of JAK/STAT pathway targeting compounds is significantly proportional to DNA methylation of the PTPRM gene promoter in pancreatic cancer cell lines (rho = 0.3, P = < 0.005)

PTPRM is an important component of STAT3 regulation with downstream effects on proliferation and metastasis in lung cancer [84] therefore we speculated whether PTPRM epigenetically deficient cancer cells could be exploited for therapeutic purposes, specifically drugs that target the JAK/STAT cell signalling pathway in other tissues. We downloaded IC_50_ concentration Z scores from GDSC2 (Genomics of Drug Sensitivity in Cancer, dataset2) database [31] for antitumour drugs that target key cancer-related cellular pathways (Fig. 5B). We identified 4 compounds, AZ960 [85], JAK8517, JAK18709 [86] and Ruxolitinib [87], that specifically target proteins in the JAK/STAT pathway and compared their drug sensitivity to *PTPRM* promoter methylation status in pancreatic cancer cell lines. We observed that drug sensitivity was significantly proportional to DNA methylation levels of the *PTPRM* gene promoter in pancreatic cancer cell lines (rho = 0.3, *P* = 0.00462) (Fig. 5C) suggesting that *PTPRM* promoter methylation profiles maybe used as a potential biomarker for clinical treatment response in pancreatic cancer patient therapy.

## Discussion

In this study, we systematically surveyed hypermethylation profiles of Protein Phosphatase Enzymes and interacting protein (PPEIP) gene promoters to highlight the role of epigenetic marks that alter transcription of tumour suppressor genes and its effects within a clinical setting. In this regard, we detected 5007 hypermethylation events in 160 PPEIP gene promoters that were absent in healthy controls (epimutations). These epimutations were detected in 539 cancer patients (84%) across 5 tissues (colorectal, oesophageal, lung, pancreatic and stomach) and may disrupt the delicate balance of protein phosphorylation in signalling networks and promote malignancies. This dysregulation of key genes is not uniform across all tissue types. By assaying epimutations in multiple tissues, we observed that stomach cancer patients accumulate the highest number of epimutations (Supplementary figure S2) while lung cancer cases produce the least. This is an interesting finding as epimutations in PPEIP have been described in both tissues, (examples include; [88, 89]) although a cumulative comparison across tissues and individuals has not been previously reported. This disparity of epimutations may relate to their function defined by regulatory signatures underlying each tissue (such as active chromatin marks H3K4me3 and H3K4me1) that accompany differences in DNA methylation patterns by tissue-specific mechanisms in malignant cells [90]. This decrease in PPEIP epimutation susceptibility (*OEDR = log2 -1.25*) in lung tumours suggests that DNA methylation is more tightly controlled in lung tumorigenesis and likely reduces the role DNA methylation plays in highly mutable signalling pathways such as PI3K-AKT, JAK/STAT and MAPK (Fig. 2) in lung cancers as compared to other tissues. The oncogenic programs that establish a favourable molecular environment for these signalling pathways likely rely on other genomic mechanisms [91]. These findings may have important clinical implications for designing treatment strategies that target PPEIP genes and their disrupted pathways.

The role of phosphatases, in particular Protein Tyrosine Phosphatases or PTP (and to a less extent, Dual Specific Phosphatases, DUSP), as tumour suppressors have been studied for decades and are emerging targets for novel technologies for oncogenic therapy [81, 92]. 57% of all cancer patients revealed epimutations in PTP genes and were overwhelmingly enriched in Colorectal Cancer probands (81%) as compared to pancreatic cancer (23%). Interestingly, epimutations in *PTPRT* were discerned in the most individuals despite very low expression in all tissues analysed (Supplementary Fig. 7A and 9B and D). This finding provides evidence that abnormal promoter hypermethylation mechanisms target certain tumour suppressors independent of their transcriptional activity. PTPRT is a negative regulator of signal transducer and activator of transcription 3 (STAT3) and therefore promoter hypermethylation events in addition to deleterious genetic mutations may be utilized as biomarkers to inform on potential neoplasm growth in multiple tissues, responsiveness to STAT3 inhibitors [93] and predictors of standard treatments against cancer [94]. In contrast, we discerned 4 PTP and 3 DUSP genes where epimutations consistently resulted in transcription silencing in all cell models (Fig. 4). All 7 genes have been implicated in several cancer-specific cellular programs as tumour suppressors and DNA methylation profiles associated with these genes may have compelling clinical implications [2]. For example, *PTPRM* epimutations were observed in the most cancer cases of the 7 genes (16%) in all 5 tissues. PTPRM is a receptor PTP and its intracellular compartment is responsible for phosphatase activity, whereas the extracellular section serves in cell–cell and cell–matrix contact [3]. STAT3 inactivation is catalysed by PTPRM dephosphorylation and leads to cancer cell death, therefore errors incurred in STAT3 dephosphorylation, such as promoter hypermethylation induced *PTPRM* transcriptional silencing, may lead to cancer initiation and progression with poor clinical prognosis [84, 95]. Furthermore, our data demonstrated that low PTPRM expression is associated with poor overall survival (Fig. 5A) and PTPRM methylation marks maybe used as biomarkers for JAK/STAT targeting anti-cancer drugs response. Additionally, the other epimutated genes showed equally intriguing roles in cancer. For example, in breast cancer, PTPN13 was reported to inhibit cancer aggressiveness by Src dephosphorylation [96] which is upregulated in tamoxifen-resistant ER-positive breast cancer patients [97], or DUSP4 repressed expression was identified as a mechanism of neoadjuvant drug chemoresistance and frequently depleted in chemotherapy refractory breast tumours [98]. Together, this highlights the role of epimutations in PTP and DUSP genes as putative cancer biomarkers for diagnosis, prognosis and treatment response.

Interestingly, we identified one example where hypermethylated promoters resulted in an increase of transcription (Supplementary Fig. 9A and C). *DNAJC6* was the second most epimutated PTP gene in cancer patients and the most in lung cancer patients (Fig. 3C). A literature search revealed DNAJC6 possesses oncogenic properties and promotes hepatocellular carcinoma (HCC) progression through induction of epithelial–mesenchymal transition. Overexpression of DNAJC6, as shown with increased promoter methylation in cancer subjects, was observed to enhance cell proliferation and invasion suggesting *DNAJC6* hypermethylation may be assayed as a putative biomarker for poor outcome in HCC [99].

## Conclusions

Overall, our data illustrates the cellular and clinical relevance of DNA hypermethylation in a subset of PPEIP. We have broadened our knowledge of PPEIP epimutations across previously undescribed genes and tissues as well as providing an insight into epimutation susceptibility and distribution and its subsequent role in tumourigenesis and treatment. Considering the clinical implications, this type of phosphatase-wide analysis of promoter hypermethylation would benefit patients with other cancer types with high levels of phosphatase activity such as brain and neuroblastomas (Supplementary Fig. 7). With advances in single cell sequencing technologies, targeting epimutations in phosphatase enzymes will reveal precise signalling pathways and transcriptional programs in clonal sub-populations of tumour cells previously undetectable with bulk technologies and vastly improve cancer therapeutics. Together, it’s seductive to posit that the emergence of epi-drugs that rehabilitate genes inactivated through epigenetic mechanisms [100] hold promise for re-activating tumour-suppressing cellular programs through genes such as *PTPRM*, that leads to improved treatment strategies and reduction in mortality of high-risk patients.

### Electronic supplementary material

Below is the link to the electronic supplementary material.

**Supplementary Material 1:** Supplementary figures 1–9

**Supplementary Material 2:** Supplementary data S1–S9

## Data Availability

Raw DNA methylation .idat files for primary tumours and healthy tissues were downloaded from the TCGA website. We incorporated 450 K DNA methylation data from healthy stomach tissue available in GEO under accession number GSE127857. Raw DNA methylation .idat files corresponding to the cancer cell lines for the 5 organs were downloaded from GEO under accession number GSE68379 and idat files for the embedded 3D cultures (organoid) representing the 5 cancer subtypes were downloaded from the GEO website under the accession number GSE144213. Finally, RNA-seq data corresponding to primary tumours and cancer cell lines was downloaded from TCGA and CCLE data repository respectively.
