# Supplementary material for: Cellular and clinical impact of protein phosphatase enzyme epigenetic silencing in multiple cancer tissues
Source: Hum Genomics. 2024 Mar 12;18:24. doi: 10.1186/s40246-024-00592-x (PMC10935810; doi:10.1186/s40246-024-00592-x)
Supplement: Supplementary file 2 — Supplementary Material 2: Supplementary data S1–S9 [file 40246_2024_592_MOESM2_ESM.pdf]

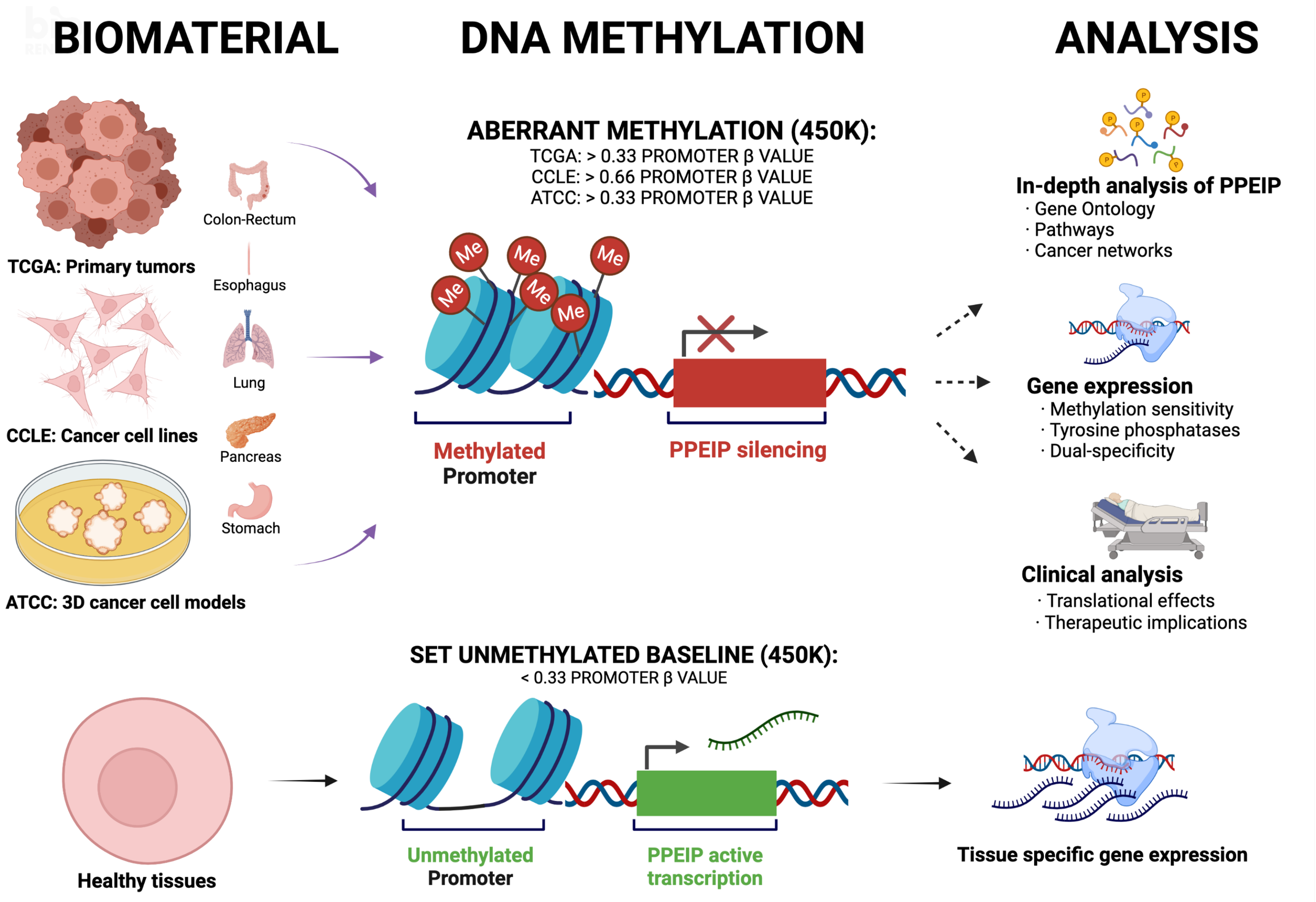

Supplementary figure 1 : Analysis workflow for DNA methylation interrogation of Protein Phosphatase Enzymes and interacting protein (PPEIP) gene promoters. As a hallmark of cancer, cancer-specific increases in gene promoter DNA methylation result in transcription repression of key cancer-related genes. Here, publicly available genome-wide DNA methylation datasets (450K) from 5 cancer subtypes (Colorectal, Esophageal, Lung, Pancreatic and stomach cancers) were examined to discern aberrant methylation profiles that disrupt PPEIP expression and their related cellular pathways. Cancer samples were analyzed from primary tumors, cancer cell lines and 3D cancer cell models. Average promoter methylation beta values of >0.33 (primary tumors and organoids) and 0.66 (cancer cell lines) were identified and their effect on transcription, cell biology and translation were reported. DNA methylation profiles from concordant healthy samples were used as a baseline to determine cancer-specific DNA methylation-induced PPEIP silencing.

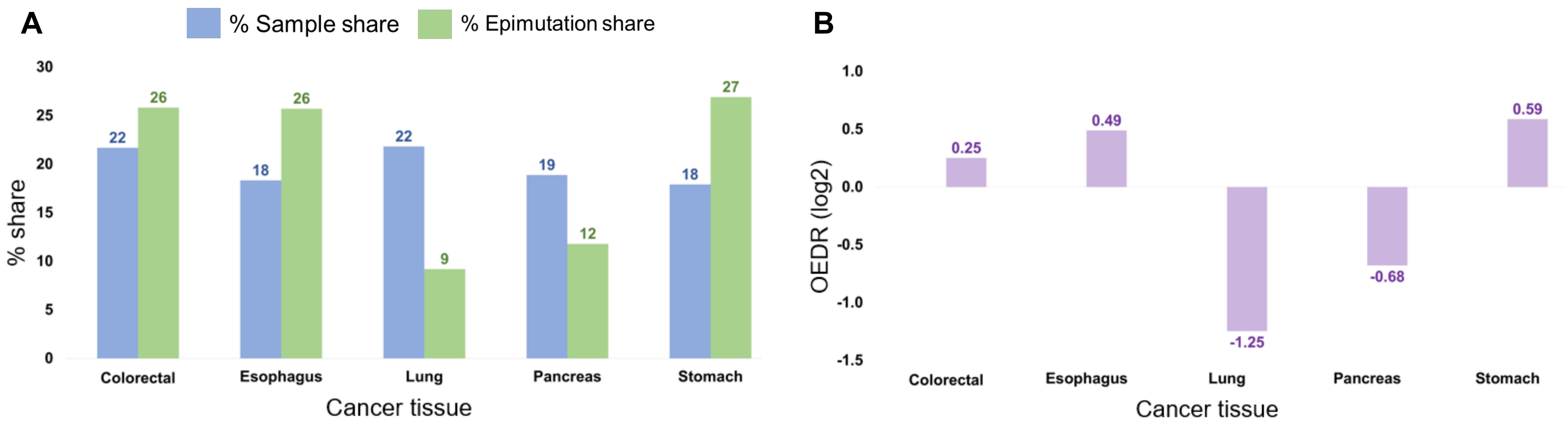

Supplementary figure 2: **Analysis of the distribution of PPEIP epimutations across cancer sample types.** A) Bar graph to show the percentage of PPEIP epimutations detected in each tissue vs total sample distribution. B) Observed Epimutation Distribution Ratio (OEDR) demonstrates the number of epimutation detected vs the expected value (0). Data reveals a disproportionate distribution of epimutations across tissue types.

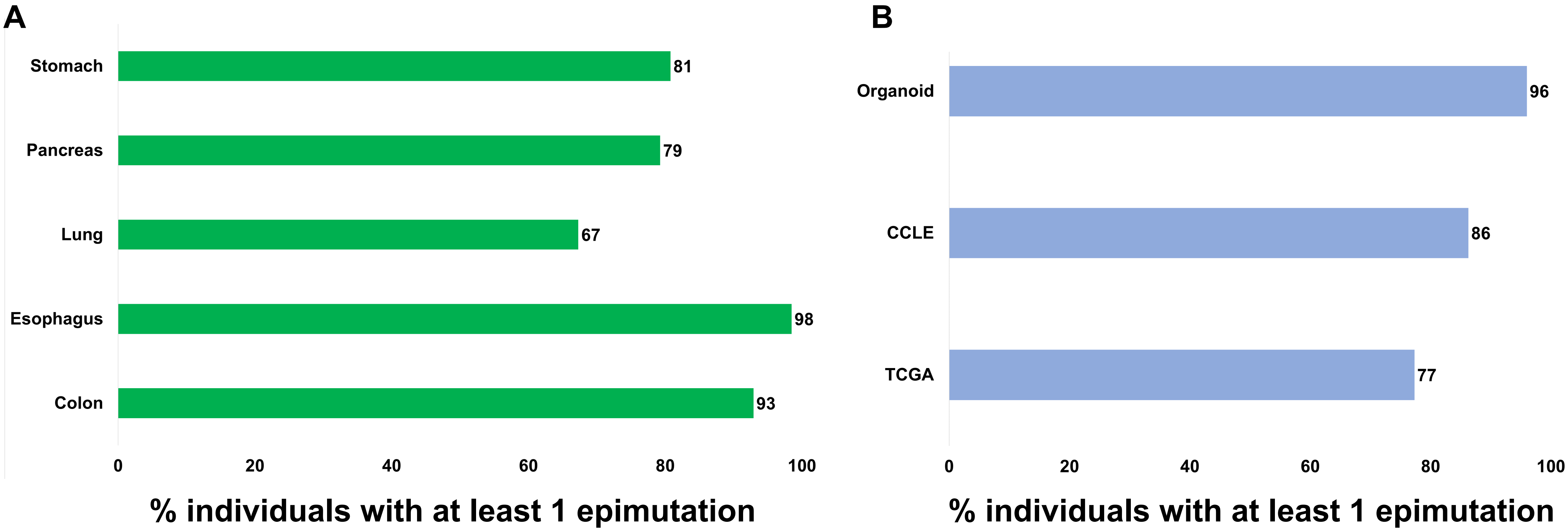

Supplementary figure 3: Breakdown of epimutation percentage detected in (A) cancer tissue types and (B) cancer cell models. Organoids represents 3D embedded cancer cell models, CCLE, cancer cell lines from the Cancer Cell Line Encyclopedia, TCGA, The Cancer Genome Atlas.

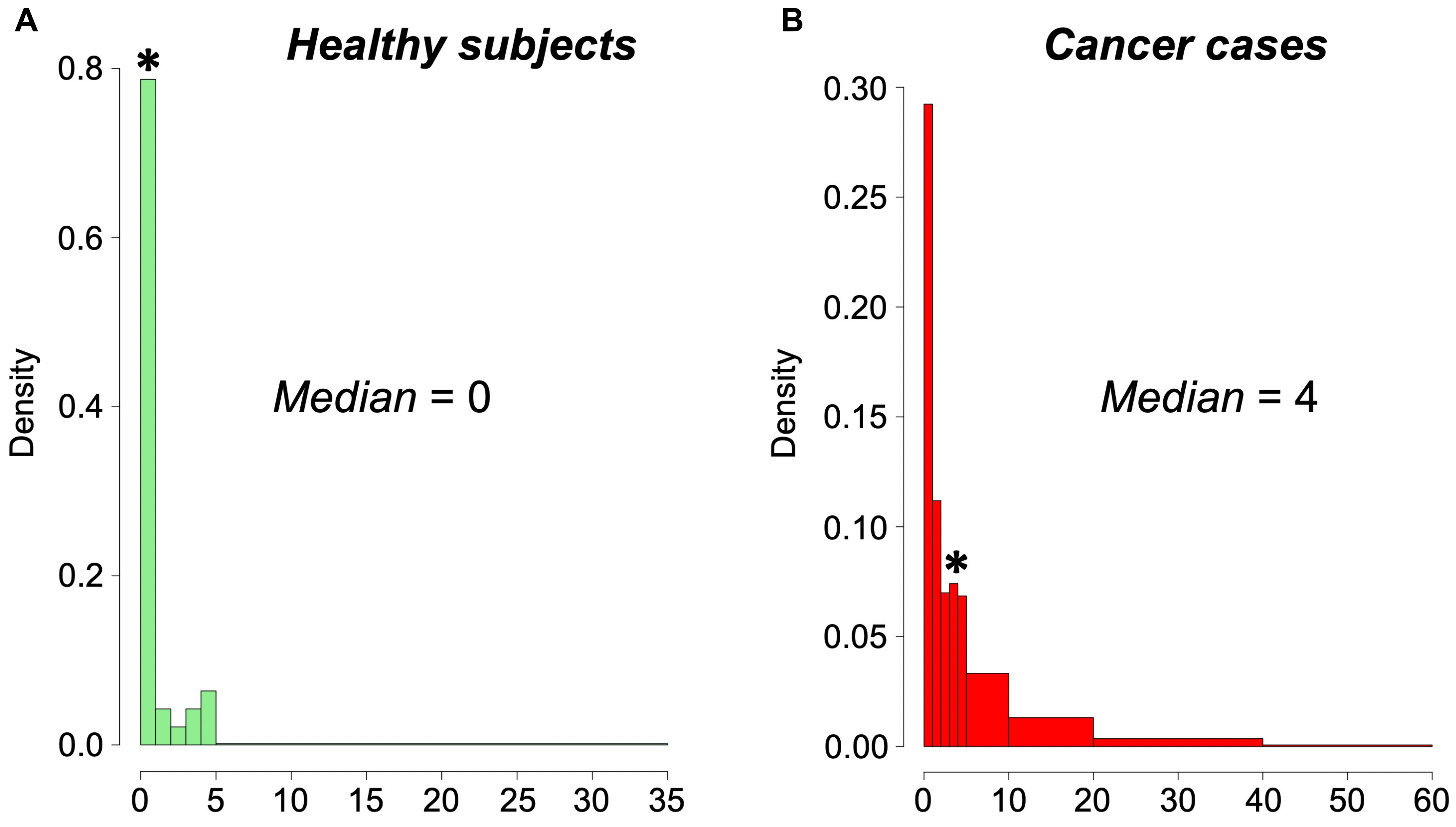

Supplementary Figure 4. Histogram showing the number of hypermethylated PPEIP promoters identified in healthy control subjects vs Cancer cases. A) The number of PPEIP epimutations in healthy individuals in the population control cohort vs control baseline samples. B) Epimutations in each individual tumor sample. Note * represents the median value of epimutations per cohort.

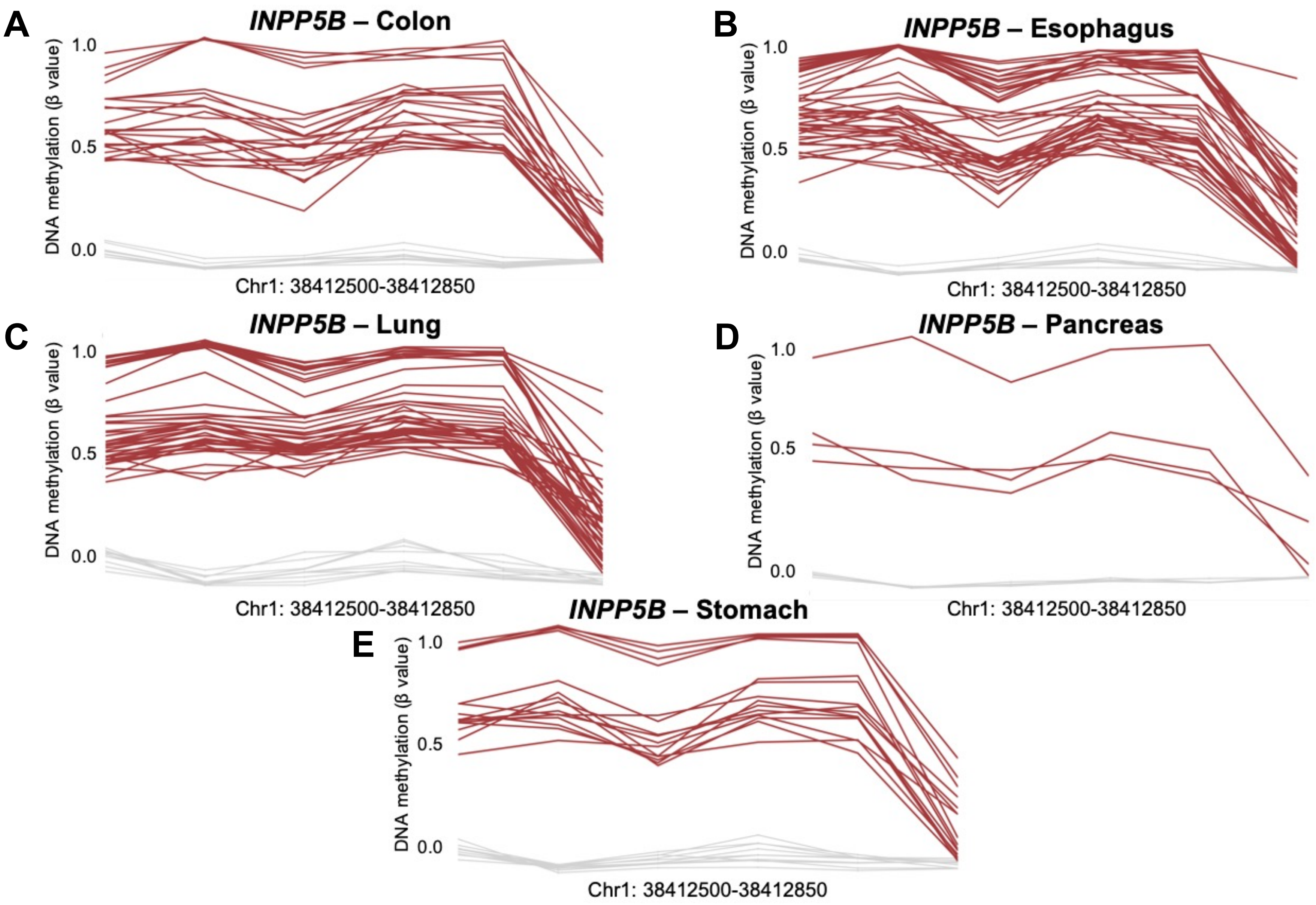

Supplementary Figure 5. Breakdown by tissue of recurrent epimutations in *INPP5B* gene promoter. A total 130 individuals were observed with cancer-associated epimutations in the *INPP5B* gene promoter. The 130 patients consisted of the following cancers; 24 = colorectal, 45 = Esophageal, 42 = Lung, 4 = Pancreatic and 15 = Stomach. Red lines present DNA methylation profiles of cancer patients with outlier epimutations and grey lines, healthy controls.

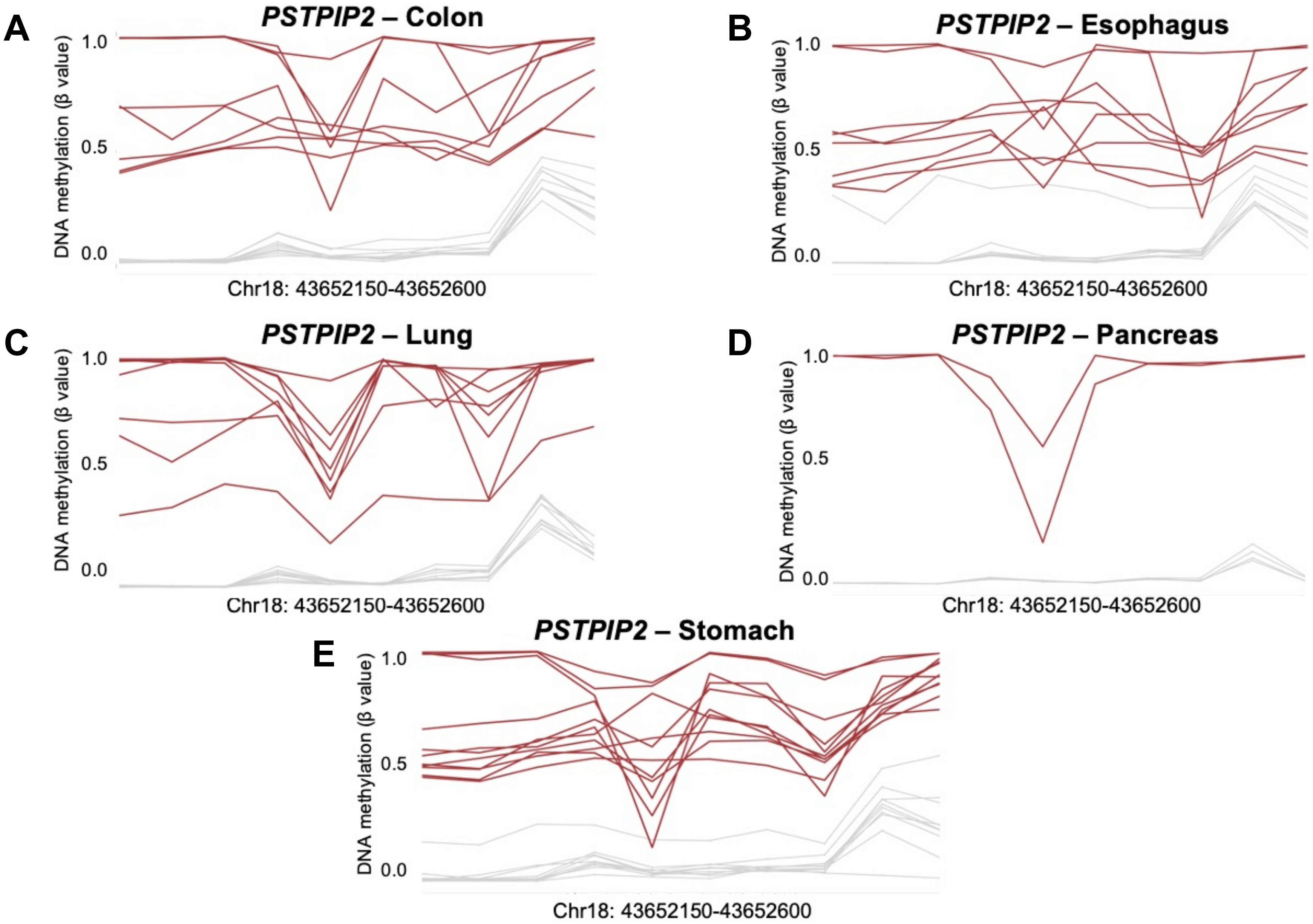

Supplementary Figure 6. Breakdown by tissue of recurrent epimutations in *PSTPIP2* gene promoter. A total 37 individuals were observed with cancer-associated epimutations in the *PSTPIP2* gene promoter. The 37 patients consisted of the following cancers; 8 = colorectal, 8 = Esophageal, 8 = Lung, 2 = Pancreatic and 11 = Stomach. Red lines present DNA methylation profiles of cancer patients with outlier epimutations and grey lines, healthy controls.

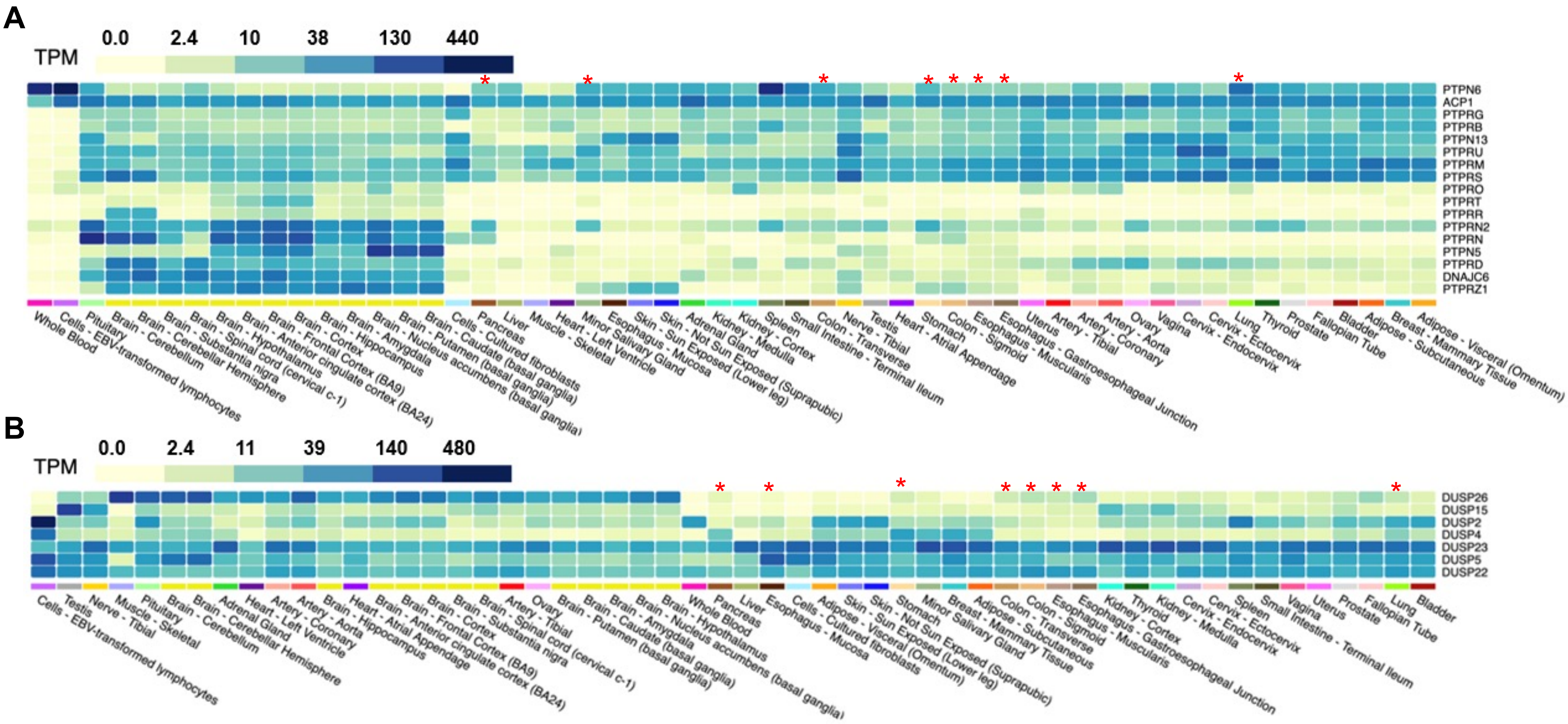

Supplementary Figure 7. Color coded visualization of PTP and DUSP expression values in healthy tissues. A) 17 PTP and B) 7 DUSP genes were discerned with epimutations in this study and their expression values in a non-cancer population from the GTEx project presented here. All values are represented as Transcripts per million (TPM). * denotes the five tissues and subtypes analyzed in this study.

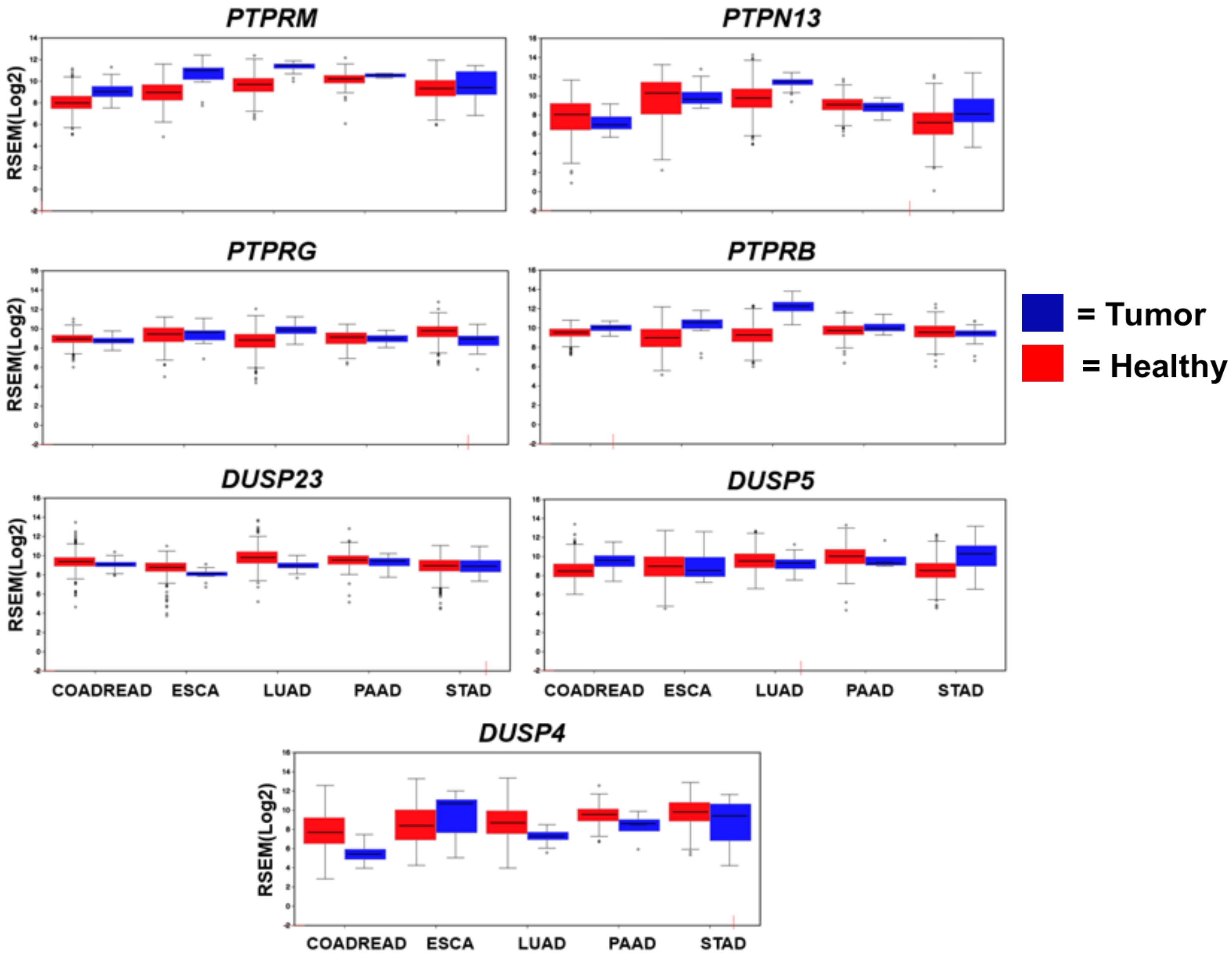

Supplementary Figure 8. Expression of ubiquitously epimutated PTP and DUSP genes comparing normal vs cancer tissues. Transcript profiles from five TCGA projects utilized in this study (COADREAD = Colon/Rectum adenocarcinoma, ESCA = Esophageal carcinoma, LUAD = Lung adenocarcinoma, PAAD = Pancreatic adenocarcinoma, and STAD = Stomach adenocarcinoma) shows the expression of highly epimutated PTP AND DUSP genes detected in multiple cell models independent of promoter methylation status. Transcript abundance shown as RSEM (RNA-Seq by Expectation Maximization) log2 values.

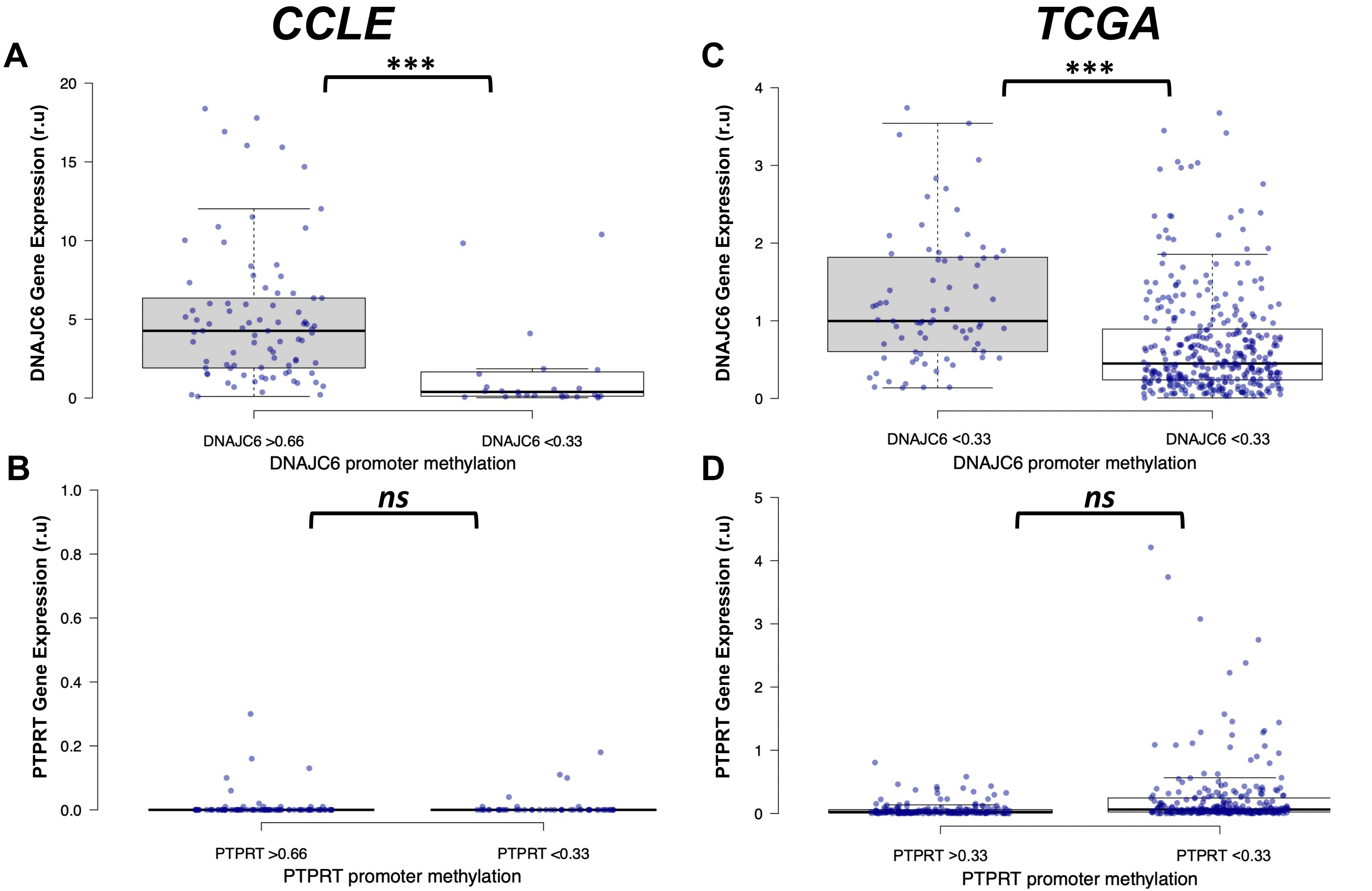

Supplementary Figure 9. Gene expression comparison of *PTPRT* and *DNAJC6* in individuals with hypermethylated promoters vs healthy controls. A and B) represent expression profiles in cancer cell lines. In the CCLE cohort, hypermethylated promoters were those with average >0.66 beta values vs healthy baseline controls (<0.33). C and D) demonstrate expression profiles in primary tumors. Hypermethylated promoters in the TCGA subset were defined as average >0.33 beta value vs baseline beta values. Each purple dot represents expression values provided by CCLE (TPM) and TCGA (FPKM) for each individual. *** *P* < 0.001, ns = not significant.
